# Characterization of the tumor microenvironment in locally advanced gastric cancer and identification of spatially predictive biomarkers associated with beneficial neoadjuvant immunochemotherapy

**DOI:** 10.3389/fimmu.2026.1823308

**Published:** 2026-05-14

**Authors:** Xiaohong Xu, Jun Fan, Li Peng, Zhengkai Xiang, Danju Luo, Chenggong Ma, Bo Huang, Xiu Nie, Xiaochuan Dong

**Affiliations:** 1 Department of Pathology, Union Hospital, Tongji Medical College, Huazhong University ofScience and Technology, Wuhan, Hubei, China; 2 Department of Thoracic Surgery, Bone and Soft Tissue Surgery, Hubei Cancer Hospital, Tongji Medical College, Huazhong University of Science and Technology, Wuhan, China

**Keywords:** biomarkers, digital spatial profiling, locally advanced gastric cancer, neoadjuvant immunochemotherapy, tumor microenvironment

## Abstract

**Background:**

Neoadjuvant immunochemotherapy (nICT) has emerged as a promising strategy for locally advanced gastric cancer (LAGC), yet clinical responses remain heterogeneous and reliable predictive biomarkers are lacking. A comprehensive dissection of the tumor microenvironment (TME) is essential to uncover determinants of therapeutic efficacy and enable precision immunotherapy.

**Methods:**

We performed digital spatial profiling (DSP) using the NanoString GeoMx platform on pretreatment endoscopic biopsies from 19 LAGC patients treated with tislelizumab plus SOX chemotherapy. Multiplex fluorescence staining (PanCK, CD45, CD68) enabled compartment-specific transcriptomic analysis of tumor center regions, immune cell infiltration area, and other stromal regions. Findings were integrated with TCGA-STAD data and validated in an independent cohort (n = 20) by immunohistochemistry (IHC) for NOTUM, SERPINA3, CD8, and FOXP3.

**Results:**

Spatial profiling revealed distinct transcriptional programs across tumor-center regions (TC), immune cell infiltration area (MA), and other stromal regions (OTHER) compartments. High tumor-intrinsic expression of NOTUM, NKD1, and SERPINA3, together with elevated CD8^+^ T cell infiltration and a reduced Treg/CD3^+^ ratio within the TME, robustly associated with major pathological response (MPR). These spatial biomarkers were orthogonally validated by IHC in an independent cohort. In TCGA-STAD, a six-gene signature (NOTUM, APOA2, SERPINA3, NKD1, GGH, BPIFB1) correlated with prolonged survival and favorable immune infiltration, with conserved immune-modulatory patterns across multiple cancer types.

**Conclusions:**

This study identifies NOTUM, SERPINA3, and CD8^+^ T cell density as spatially resolved, clinically actionable predictors of nICT response in LAGC. Our findings underscore the power of spatial TME interrogation to uncover novel biomarkers and guide personalized immunotherapeutic strategies.

## 1 Introduction

Gastric cancer (GC) ranks as the fifth most common malignancy and the fourth leading cause of cancer-related mortality worldwide (1–3). Owing to its atypical early symptoms and insufficient screening systems, approximately 76.5% of patients are diagnosed at the locally advanced stage (LAGC), defined as tumor invasion beyond the submucosa without distant metastasis, corresponding to TNM stages T2–4aN0–3M0 (Ib–IIIc) (4–6). Radical surgery remains the primary treatment for LAGC, yet outcomes of surgery alone or postoperative adjuvant chemotherapy are suboptimal. Neoadjuvant therapy can reduce tumor stage, decrease tumor burden, eliminate micrometastases, and improve resection rates, making it an integral part of multimodal treatment (7, 8).

In recent years, immunotherapy has markedly transformed the management of advanced gastric cancer. PD-1 inhibitors combined with chemotherapy have been approved by the U.S. FDA and China NMPA as first-line treatments and are recommended by international guidelines (7, 9). Tislelizumab, a domestically developed PD-1 inhibitor, has demonstrated favorable efficacy and safety in patients with advanced GC owing to its unique structural modifications (10–12). Tislelizumab has also demonstrated efficacy in the neoadjuvant setting. In a phase II trial, its combination with the SOX regimen (oxaliplatin and tegafur) yielded a pathological complete response (pCR) rate of 23.8% and a major pathological response (MPR) rate of 61.9% in patients with LAGC, along with a manageable safety profile (13, 14). The results of the global phase III RATIONALE-305 trial demonstrated that compared with chemotherapy alone, tislelizumab plus chemotherapy significantly improved overall survival (OS) (median OS 15.0 vs. 12.9 months; HR 0.80; P = 0.001) (15, 16). However, treatment responses to neoadjuvant immuno-chemotherapy (nICT) exhibit marked heterogeneity. Only a limited subset of patients achieve a pCR or MPR, while approximately 15% derive minimal clinical benefit (17, 18). Therefore, the identification of predictive biomarkers for nICT response is critically needed to facilitate personalized treatment strategies.

Accumulating evidence suggests that dynamic changes in the tumor microenvironment (TME), particularly in immune cell composition, are closely associated with nICT outcomes (12, 19). Multiomics analyses, such as whole-exome sequencing, bulk transcriptomics, and single-cell RNA sequencing, conducted in comparisons between MPR and non-MPR patients have demonstrated that increased infiltration of cytotoxic T cells and natural killer (NK) cells, together with a decrease in regulatory T cell (Treg) abundance, are positively associated with treatment response (14, 16, 17). Although the role of the TME in GC prognosis and therapeutic response has been extensively studied, its spatial context in LAGC remains incompletely understood. Spatial characterization of tumor cells and their surrounding TME is critical for predicting cancer outcomes. Direct analysis of clinical samples from LAGC patients allows for comprehensive TME profiling and more accurate identification of molecular determinants of treatment efficacy. Emerging technologies such as digital spatial profiling (DSP) provide powerful tools for this purpose. The NanoString DSP platform enables comprehensive spatial characterization of the tumor immune microenvironment (20–22), allowing high-plex quantification of mRNA and protein expression within specific regions of interest (ROIs) or areas of illumination (AOIs) in formalin-fixed paraffin-embedded (FFPE) tissues. It has successfully been applied in spatial biomarker discovery across multiple cancer types (23–27). Additionally, multiplex immunofluorescence (mIF) facilitates the simultaneous detection of multiple markers on a single tissue section and is widely used in TME studies (28–30).

In this study, DSP and mIF were integrated to investigate a cohort of LAGC patients who received neoadjuvant treatment with tislelizumab combined with the SOX regimen (oxaliplatin plus tegafur). The cohort included both MPR and non-MPR patients. Using pre-nICT endoscopic biopsy samples, we performed spatial multiomics profiling via DSP to systematically characterize the architecture of the TME and identify biomarkers predictive of sensitivity to nICT. Our results demonstrated that the expression levels of NOTUM and SERPINA3 in TC regions, together with the infiltration densities of CD8^+^ T cells and Tregs in OTHER regions, constitute a novel composite biomarker for predicting nICT response.

## 2 Materials and methods

### 2.1 Ethics statement

Written informed consent was obtained from all patients, and the study was conducted in accordance with the ethical guidelines of the Declaration of Helsinki. This study was approved by the Medical Ethics Committee of Union Hospital, Tongji Medical College, Huazhong University of Science and Technology (No (2022).0782-01).

### 2.2 Patient cohorts

A retrospective analysis was conducted on pretreatment tumor biopsy specimens from 41 patientswith LAGC collected at Union Hospital, Tongji Medical College, Huazhong University of Science and Technology, between January 2021 and August 2025. All LAGC patients met the TNM stage of T2-4AN0-3M0 (IB-IIIC stage). No patients received any other special treatments before biopsy. Following biopsy, all patients underwent three cycles of combination therapy with tislelizumab, oxaliplatin, and tegafur, followed by surgical resection in accordance with the NCCN guidelines. Further details regarding the inclusion and exclusion criteria, clinicopathological characteristics, and treatment regimens are provided in **Supplementary Table S1**, **Supplementary Tables S2**, **S3**. The patients were randomly divided into two groups: the first group was designated for tumor microenvironment characterization and identification of biomarkers associated with the response to nICT (discovery cohort, n=19), and the second group was designated for validation (validation cohort, n=20). In addition, a bioinformatics analysis was conducted on a cohort of gastric adenocarcinoma patients from The Cancer Genome Atlas (TCGA) (TCGA cohort) to explore the clinical value of potential biomarkers.

### 2.3 Tumor regression grade assessment

The tumor regression grade (TRG) was evaluated according to the American Joint Committee on Cancer (AJCC) 8th edition criteria, which classify treatment response based on the extent of residual tumor and fibrotic changes in the surgical specimen. This four-tier system includes the following: TRG 0 (complete response, no viable tumor cells), TRG 1 (moderate response, minimal residual cancer in the form of small clusters or single cells), TRG 2 (minimal response, residual cancer predominated by fibrosis), and TRG 3 (poor response, minimal or no tumor loss with extensive residual cancer). For analytical purposes, patients with a TRG of 0 or 1 were categorized as major responders, while those with a TRG of 2 or 3 were considered nonresponders.

### 2.4 Digital spatial profiling

Digital spatial profiling (DSP) was performed using an established method developed by NanoString Technologies. FFPE tissue sections were hybridized with UV-cleavable barcode-labeled RNA *in situ* hybridization probes to capture and analyze mRNA expression profiles. Concurrently, fluorescence labeling was applied for pan-cytokeratin (PanCK) and common leukocyte antigen (CD45) to distinguish tumor regions and immune cell infiltrates, and CD68 fluorescence labeling was used to identify macrophages. The labeled slides were scanned using a GeoMx^®^ instrument, and regions of interest (ROIs) were selected based on immunofluorescence imaging and were reviewed and confirmed by a pathologist from Union Hospital, Wuhan. Automated segmentation via customized UV illumination templates was used to generate four distinct AOIs: tumor-center regions (PanCK^+^CD45^-^CD68^-^; TCs), immune cell infiltration area (PanCK^-^CD45^+^CD68^-^ and PanCK^-^CD45^-^CD68^+^; MA), and other stromal regions (PanCK^-^CD45^-^CD68^-^; OTHER). Finally, the released photocleavable barcodes from each AOI were collected and quantitatively analyzed using sequencing technology.

### 2.5 Spatial transcriptomic data analysis

The GeoMx^®^ NGS Pipeline was employed to convert raw sequencing data in FASTQ format into digital count conversion (DCC) files. Stringent quality control (QC) procedures were applied, including assessments of technical signals, background noise, probe performance, and normalization. AOIs with alignment rates of reads to the template sequence less than 80% were excluded. Technical background evaluation incorporated three metrics: nontemplate control (NTC) counts, negative probe counts, and AOI-specific parameters. Specifically, AOIs with NTC counts exceeding 1000 were discarded to mitigate potential template contamination. The threshold for negative probe counts was set at 4 to establish the baseline technical noise level. AOI QC criteria required a nucleus count >20 or an area >1600 µm² (25). To minimize inter-AOI variation, the data were subjected to size factor correction and normalized to the nucleus count and area. High-quality data were subsequently processed using Quantile 3 normalization. Dimensionality reduction was performed using uniform manifold approximation and projection (UMAP) and t-distributed stochastic neighbor embedding (t-SNE). Differential gene expression analysis was conducted using edgeR (v3.34.0), with significance defined as a false discovery rate (FDR) < 0.05 and a |log_2_(fold change)| > 1. Pseudobulk RNA sequencing data were generated by averaging expression values across all AOIs per sample using the formula (X_1_ + X_2_ +… + X_n_)/N, where X represents gene expression and N is the number of AOIs. Based on the differential expression results, a composite six-gene signature was further constructed using genes that were consistently enriched in responders across the pseudobulk and region-specific spatial analyses. Candidate genes were selected using the thresholds FDR < 0.05 and |log2FC| > 1, and those showing robust association with response across multiple spatial compartments were prioritized. The final signature included NOTUM, APOA2, SERPINA3, NKD1, GGH, and BPIFB1. For downstream analyses, the six-gene signature score was defined as the mean normalized expression of the six genes and was subsequently evaluated in the TCGA-STAD cohort for its prognostic and immune-correlation relevance. Pathway enrichment analysis was performed using KEGG database annotations, with an FDR-adjusted p value < 0.05 considered to indicate statistical significance. Immune cell abundances within the TME were inferred using SpatialDecon deconvolution analysis. The feature scores were calculated as ΣX_i_/n, where X_i_ denotes the normalized expression value of each feature gene and n represents the total number of feature genes. Finally, single-sample gene set enrichment analysis (ssGSEA) was applied to the gene expression matrix to evaluate group differences across immune response, innate immunity, cellular functions, signaling pathways, and metabolic characteristics.

### 2.6 Immune cell signature analysis

The abundance of 24 immune cell types—including 18 T cell subsets and 6 of other immune cells (B cells, NK cells, monocytes, macrophages, neutrophils, and dendritic cells)—was estimated using ImmuCellAI (Immune Cell Abundance Identifier), a computational tool designed for deconvoluting immune cell compositions from gene expression data (including RNA-seq and microarray data). The resulting abundance values were normalized to zero mean and unit variance (Z score) and visualized via a heatmap. To enhance the clarity of the visualization, normalized values were Winsorized at a mean ± 2 standard deviations, ensuring that the color distribution represented the majority of the data (approximately 99% of the values fell within this range). A distance matrix was constructed using Ward’s minimum variance method (Ward.D2) for hierarchical clustering, with smaller distances indicating greater similarity between AOIs/ROIs. For comparative analyses between groups, appropriate nonparametric statistical tests were applied on the basis of the experimental design: the Wilcoxon rank-sum test was used for comparisons between two independent groups, and the Kruskal–Wallis test was employed for multigroup comparisons.

### 2.7 Immunohistochemical staining

IHC staining was performed on pretreatment tumor biopsy specimens from patients with LAGC. FFPE tissue sections were processed using a standardized protocol. Briefly, the sections were dewaxed in xylene and rehydrated through a graded ethanol series. Antigen retrieval was carried out via microwave heating in the appropriate buffer, after which endogenous peroxidase activity was blocked with 3% hydrogen peroxide. After they were incubated with goat serum to reduce nonspecific binding, the sections were incubated for 3 h at room temperature with the following primary antibodies: anti-NOTUM (ab106448; Abcam; 1:2000), anti-SERPINA3 (ab205198; Abcam; 1:2000), anti-CD3 (ab16669; Abcam; 1:500), anti-CD8 (ab217344; Abcam; 1:500), and anti-FOXP3 (ab20034; Abcam; 1:500). A horseradish peroxidase (HRP)-conjugated secondary antibody was subsequently applied, and antigen–antibody complexes were visualized using 3,3′-diaminobenzidine (DAB) as the chromogenic substrate. Finally, the sections were counterstained with hematoxylin, dehydrated through a graded alcohol series and xylene, and mounted with neutral resin. All the stained sections were evaluated microscopically by two senior pathologists. Appropriate positive and negative controls were included in each staining experiment. To quantify protein expression, digital images of stained sections were captured under identical microscope settings. The IHC staining intensity was analyzed using ImageJ software (National Institutes of Health, Bethesda, MD, USA). For each section, representative fields were selected, and the integrated optical density (IOD) and/or mean optical density (MOD) of the positive staining area was measured. The staining score was calculated based on the extent and intensity of immunoreactivity, and the average value from multiple fields was used for statistical analysis. All evaluations were performed in a blinded manner to minimize observer bias.

### 2.8 Statistical analysis

Statistical analyses were performed to assess group differences and correlations. The Wilcoxon rank-sum test was used to compare continuous variables between two groups, while the Pearson correlation coefficient was used to evaluate linear relationships between variables. Survival analysis was conducted using the Kaplan–Meier method, and differences between survival curves were compared using the log-rank test. Heatmaps were generated using the R package “Complex Heatmap”. A two-sided p value of less than 0.05 was considered to indicate statistical significance. Receiver operating characteristic (ROC) curve analysis was performed to evaluate the diagnostic performance of the selected markers in distinguishing MPR from non-MPR patients. The optimal cut-off value was determined based on the maximum Youden index (sensitivity + specificity − 1). The area under the curve (AUC), sensitivity, and specificity were calculated accordingly.

## 3 Results

### 3.1 Spatial transcriptomic analysis of LAGC using DSP

**Figure 1:**
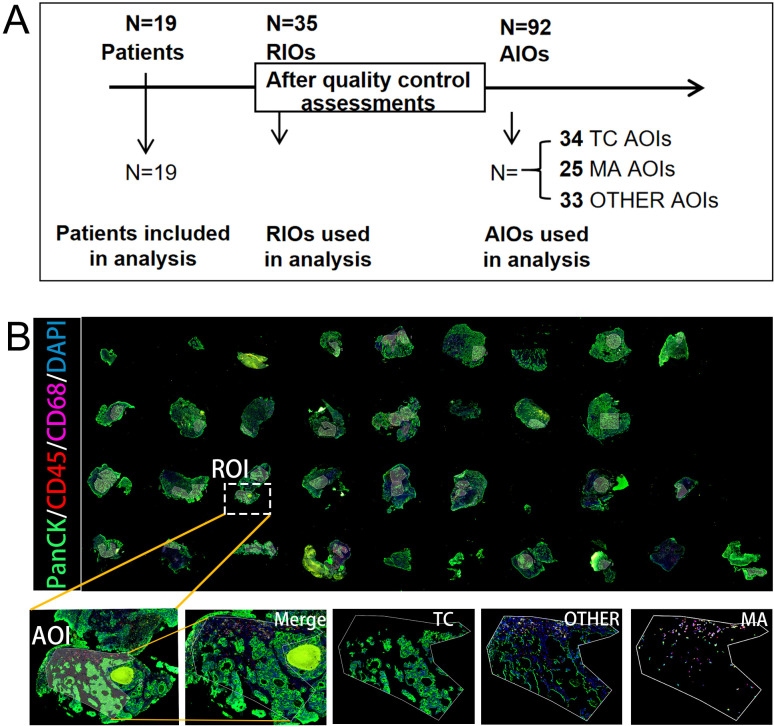
Spatial transcriptomic profiling of pretreatment LAGC biopsies using digital spatial profiling (DSP). **(A)** Workflow of sample processing and analysis. **(B)** Representative multiplex immunofluorescence (mIF) images of biopsy sections stained for PanCK (tumor, green), CD45 (leukocytes, red), CD68 (macrophages, rose red), and DAPI (nuclei, blue). The white dashed box indicates a selected ROI. Scale bar, 200 µm.

Digital spatial profiling (DSP) was performed on biopsy samples from 19 patients with LAGC usingthe NanoString GeoMx Cancer Transcriptome Atlas, which encompasses more than 18,000 genes targetinghundreds of cancer and tumor microenvironment-related pathways. The clinical and pathologicalcharacteristics of all the patients are summarized in **Supplementary Tables S2**, **S3**. Multiplex immunofluorescence staining for PanCK (green), CD45 (red), and CD68 (rose red) was applied to distinguish tumor-center regions (PanCK^+^CD45^-^CD68^-^; TCs), immune cell infiltration area (PanCK^-^CD45^+^CD68^-^ and PanCK^-^CD45^-^CD68^+^; MA), and other stromal regions (PanCK^-^CD45^-^CD68^-^; OTHER). Fluorescence images were acquired via microscopy to guide the selection of regions of interest (ROIs), which were further subdivided into 1–3 AOIs (**Figure 1**). After stringent quality control, each AOI was confirmed to meet the criteria for tissue quality and experimental validity (**Supplementary Figure 1A**). Subsequently, gene filtering was applied by retaining only genes detected above the limit of quantification in at least 20% of AOIs. This resulted in 12,970 genes retained for downstream analysis, following the removal of 5,707 lowly expressed genes from an initial total of 18,677 (**Supplementary Figure 1B**). The expression data were then normalized using the third quantile (Q3) normalization method (**Supplementary Figure 1C**). After stringent quality control, a total of 35 ROIs and 92 AOIs were included for analysis: 34 TC AOIs, 25 MA AOIs, and 33 OTHER AOIs (**Figure 1A**).

### 3.2 Characterization of the LAGC TME via spatial transcriptomics

**Figure 2:**
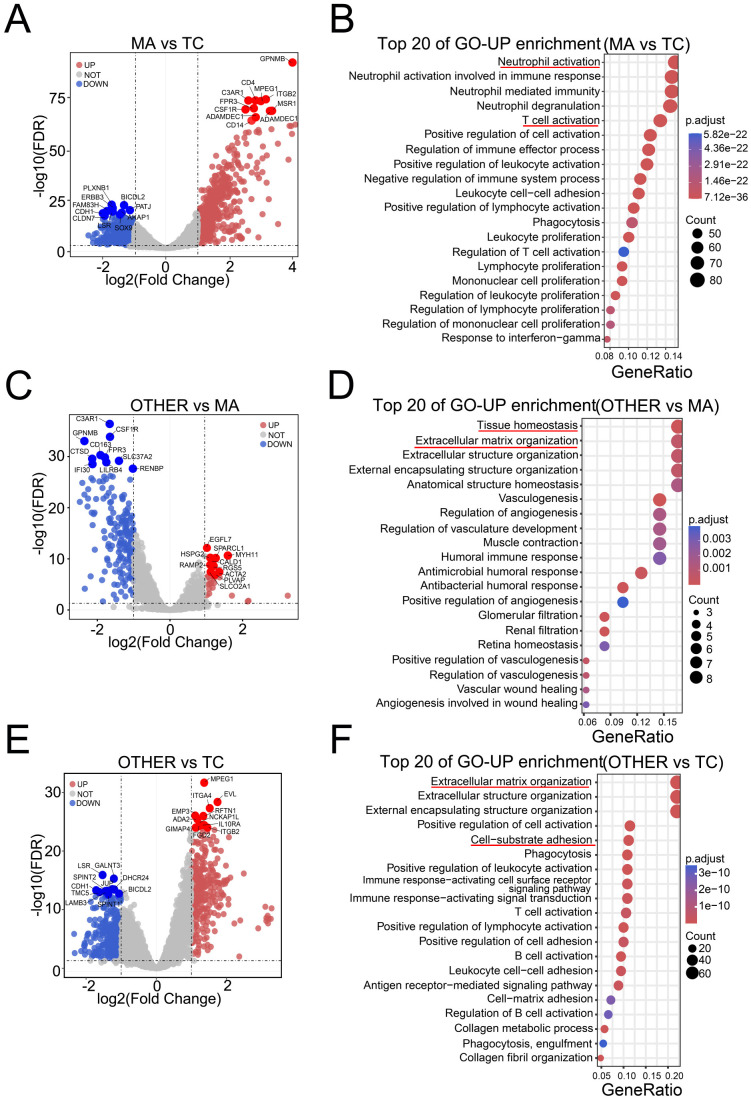
Spatial compartmentalization of the LAGC TME reveals distinct transcriptional programs. **(A)** Volcano plot showing differentially expressed genes (DEGs) between MA and TC regions. **(B)** Gene Ontology (GO) enrichment analysis of upregulated DEGs in MA regions. **(C)** Volcano plot of DEGs between the OTHER and MA regions. **(D)** GO terms enriched among upregulated DEGs in OTHER regions. **(E)** Volcano plot of DEGs between the OTHER and TC regions. **(F)** GO enrichment of upregulated DEGs associated with OTHER compared with TC.

Life Science Identifiers (LSIDs) for ZOOBANK registered names or nomenclatural acts should be listed in the manuscript before the keywords with the following format:

To decipher spatial heterogeneity in the LAGC TME, we compared gene expression profiles and pathway enrichment across functionally distinct regions. Among the immune-enriched regions (MAs) and tumor regions (TCs), 1000 differentially expressed genes (DEGs) were identified, with 521 genes showing upregulated expression and 479 genes showing downregulated expression in MAs. The upregulated genes in MAs included immune-related markers such as GPNMB, CD163, and CD4, whereas the downregulated genes (e.g., CDH1 and ERBB2) were associated with epithelial structure and signaling (**Figure 2A**).Gene Ontology (GO) analysis revealed that MA- associated upregulated DEGs were enriched in immune activation processes, including neutrophil activation and T-cell activation (**Figure 2B**), whereas MA- associated downregulated (i.e., TC-associated upregulated) DEGs were linked toepithelial development and junction assembly (**Supplementary Figure 2A**).

Comparisons between the OTHER and MA regions revealed 268 DEGs (48 upregulated and 220 downregulated in the OTHER region). OTHER-associated upregulated genes such as SPARCL1 and MYH11 were related to stromal structure and angiogenesis, whereas downregulated genes such as C3AR1 and CD163 were associated with the immune response (**Figure 2C**). The GO terms for the OTHER-associated upregulated DEGs included tissue homeostasis and extracellular matrix organization (**Figure 2D**), whereas OTHER-associated downregulated (i.e., MA-associated upregulated) DEGs wereenriched in neutrophil activation and antigen presentation (**Supplementary Figure 2B**). Furthermore, 650 DEGs were identified between the OTHER and TC regions, with 367 upregulated and 283 downregulated genes in the OTHER region. Upregulated genes such as VIM and SPARCL1 were implicated in extracellular matrix and immunomodulation, whereas downregulated genes such as CDH1 and LSR were involved in epithelial structure and differentiation (**Figure 2E**). The results of functional enrichment supported these findings, with OTHER-associated upregulated DEGs associated with cell–matrix adhesion and immune activation (**Figure 2F**), and OTHER-associated downregulated (TC-associated upregulated) DEGs enriched in epithelialdevelopment and cell junction pathways (**Supplementary Figure 2C**).

### 3.3 DSP identifies biomarkers linked to major pathological response after nICT

**Figure 3:**
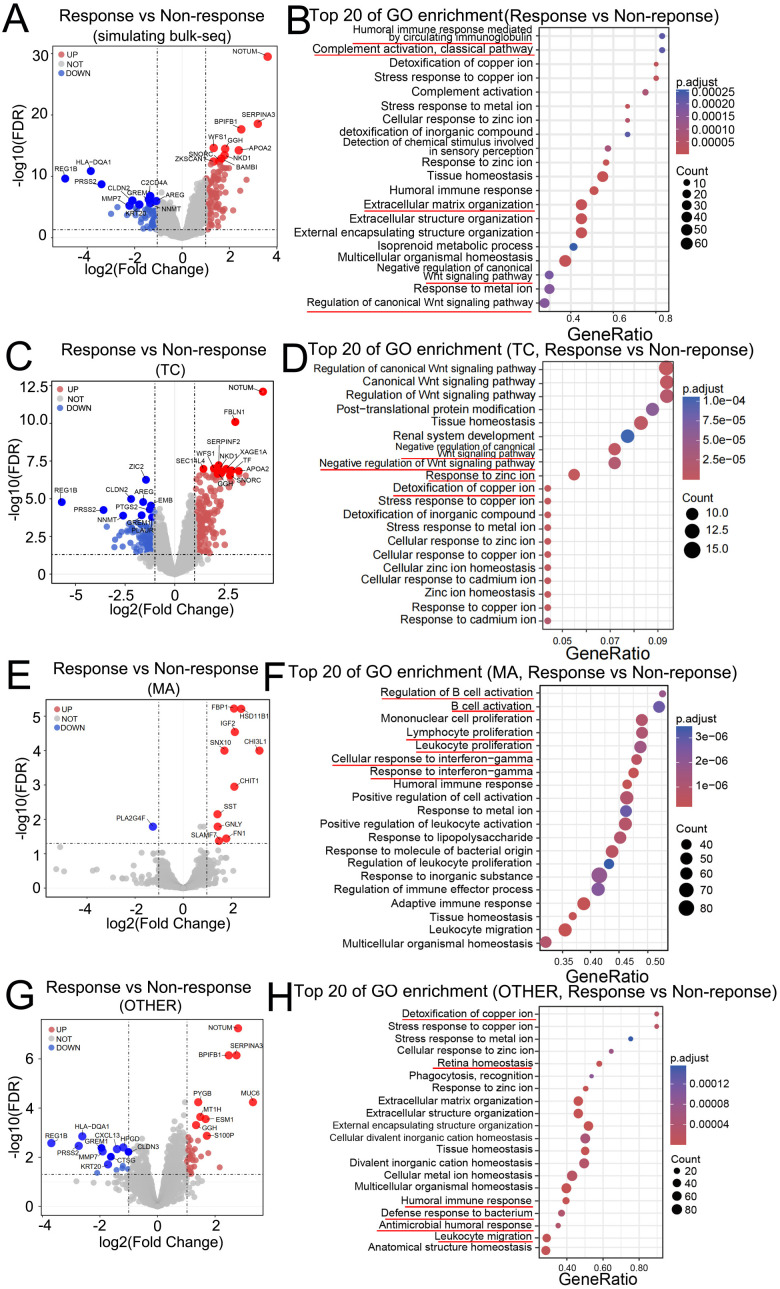
Spatially resolved biomarkers associated with major pathological responses to nICT. **(A)** Volcano plot of DEGs between responders and nonresponders in pseudobulk data (simulating bulk RNA−seq data). **(B)** Gene Ontology (GO) enrichment of the DEGs from **(A, C, E, G)** Volcano plots of DEGs between responders and nonresponders within the TC **(C)**, MA **(E)**, and OTHER regions **(G)**. **(D, F)** Top enriched GO terms for DEGs in the TC **(D)** and MA regions **(E)**. **(G)** Top enriched GO terms for DEGs in OTHER regions. Red/blue points or bars indicate up−/downregulated genes in responders.

We next investigated the association between spatially resolved gene expression and treatment response in LAGC patients receiving nICT. Based on TRG, eight patients were classified as MPRs (TRG 0, responders), and eleven were classified as nonMPRs (TRG 2–3, nonresponders). Initially, all the AOIs from each tumor sample were averaged to derive a synthetic value simulating bulk sequencing data. The responders exhibited differential expression of genes such as NOTUM, SERPINA3, APOA2, BPIFB1, NKD1, and GGH (**Figure 3A**), and were enriched in gene expression related to the humoral immune response, the WNT pathway, and the ECM-receptor interaction (**Figure 3B**). Region-specific analysis revealed distinct response signatures. In tumor regions (TCs), responders had elevated expression of NOTUM, FBLN1, APOA2, NKD1, and GGH (**Figure 3C**)—partially overlapping with bulk signatures—which supports the central role of these genes in treatment efficacy. Expression of these genes correlated with WNT inhibition and metal ion homeostasis (**Figure 3D**) (31–34). To further delineate the functional pathways linked to nICT response within tumor regions, we performed Gene Set Enrichment Analysis (GSEA) based on GO terms. The analysis revealed a significant enrichment of the WNT signaling pathway in responders (**Supplementary Figure 4A**). In immune-rich zones (MAs), the expression of response-associated genes (e.g., FBP1, CHI3L1, IGF2, and CHIT1; Figure. 3E) was associated with B cell activation, the interferon-γ response, and leukocyte proliferation (**Figure 3F**). GSEA of MA specific GO terms further elucidated these immune activation pathways (**Supplementary Figure 4B**). In the stromal region (OTHER), the differentially expressed genes included NOTUM, SERPINA3, and BPIFB1 (**Figure 3G**). These upregulated signaling pathways primarily involved the maintenance of metal ion and tissue homeostasis, extracellular matrix remodeling, and the activation of innate and humoral immune defenses against bacteria (**Figure 3H**). Complementary GSEA performed on OTHER-enriched GO terms further strengthened thesespecific pathway activities (**Supplementary Figure 4C**). Spatial expression patterns of these response-associated genes across individual AOIsfurther illustrated their inter-AOI heterogeneity and compartment-specific distribution (**Supplementary Figure 5**).

### 3.4 Spatial immune signatures correlate with nICT response

**Figure 4:**
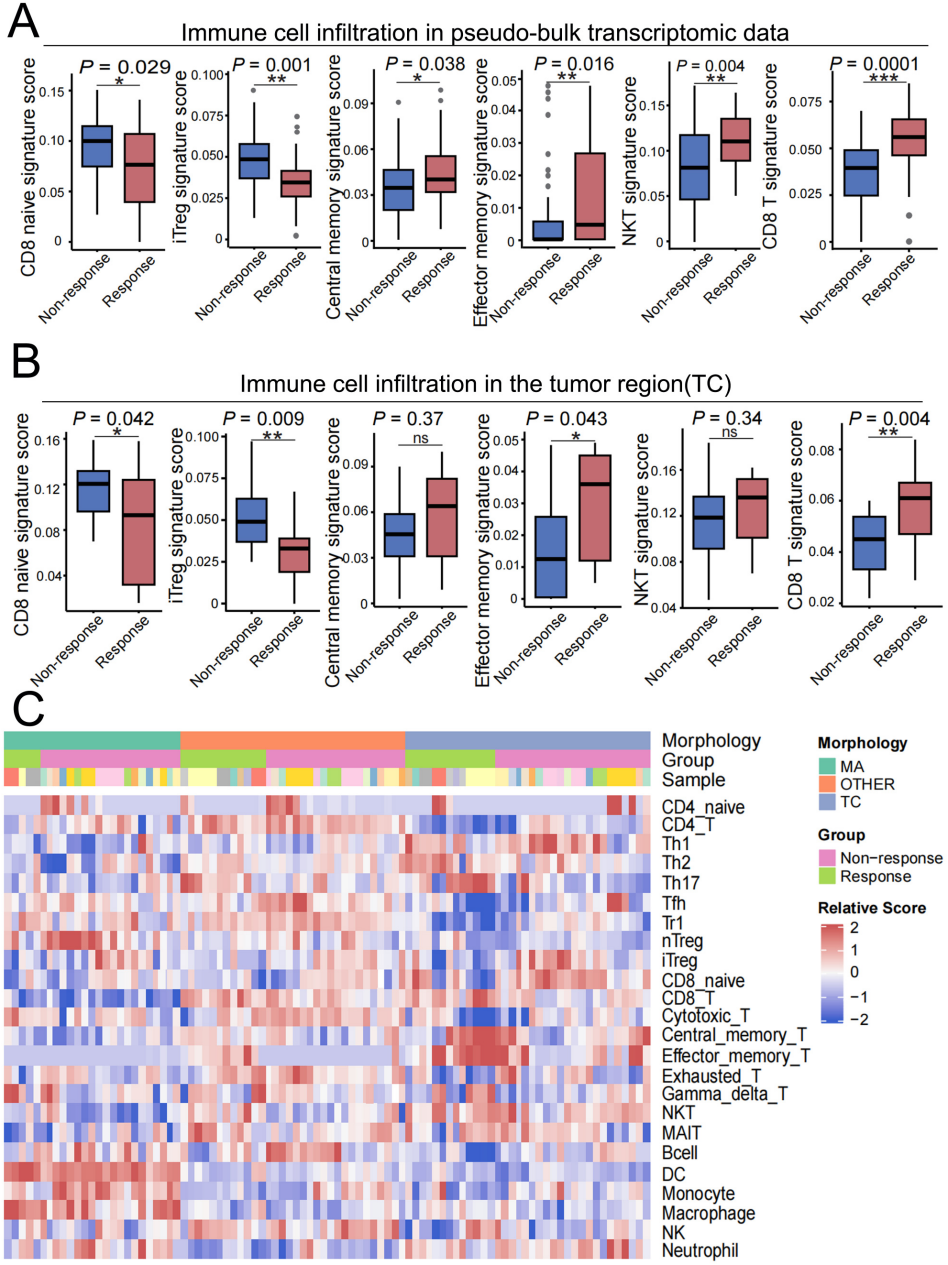
Spatial immune signatures correlate with nICT response. **(A)** Comparison of immune-related signature scores, calculated from normalized expression values of predefined marker genes, between responders and nonresponders in pseudobulk transcriptomic data. **(B)** Comparison of the same immune-related signature scores within the tumor-enriched region (TC). **(C)** Heatmap of the relative abundance of 24 immune cell subtypes across all AOIs, grouped by morphological region (TC, MA, or OTHER) and response status, as estimated by ImmuCellAI. The color scale indicates the Z score. (ns, not significant; *P* < 0.05, *; *P* < 0.01, **; *P* < 0.001, ***).

To evaluate the association between immune contexture and treatment response, we first compared immune-related signature scores derived from normalized expression values of predefined marker gene sets. According to the pseudobulk transcriptomic data, compared with nonresponders, responders had significantly elevated signature scores for CD8^+^ T cells, activated NK cells, central memory T cells, and effector memory T cells, whereas Treg scores were markedly lower (**Figure 4A**). These findings suggest that a preexisting active immune microenvironment—characterized by increased cytotoxic and memory T cell infiltration and decreased immunosuppressive Treg presence—is associated with a favorable pathological response. We further investigated the spatial distribution of these immune subsets within tumor-enriched regions (TCs). Consistent with the results of the bulk analysis, responders had significantly higher signature scores for CD8^+^ T cells, activated NK cells, and memory T cells within TC regions (**Figure 4B**). Notably, Treg scores were significantly lower in the TCs of responders, indicating aspatially nuanced immune landscape in which effector populations are enriched while regulatory T cells are relatively reduced within the tumor cores of responsive patients. To provide a comprehensive view of immune functional states, we compared the ssGSEA scores of immune-related pathways between the response groups (**Supplementary Figure 3A**). The responders demonstrated significant downregulation of allograft rejection, complementactivation, inflammatory response, and interferon-alpha/gamma signaling pathways, suggesting a lessinflamed but more organized antitumor immune milieu prior to therapy (**Supplementary Figure 3B**).

To further capture the global architecture of the tumor-immune microenvironment, we next applied ImmuCellAI to estimate the relative abundance of 24 immune cell subtypes across all profiled AOIs. The resulting Z score-normalized abundance matrix was visualized as a spatially resolved heatmap grouped by morphological region (TC, MA, and OTHER) and response status (**Figure 4C**) (35). This analysis revealed marked spatial heterogeneity in immune composition across niches and between response groups. Notably, TC regions in responders displayed a relative enrichment of cytotoxic and memory T cell signatures, whereas OTHER regions exhibited a more diverse immune infiltrate. This spatial atlas underscores the compartmentalized nature of immune responses and complements the quantitative signature analyses, providing a visual integration of the cellular ecosystem that underlies differential treatment outcomes. Collectively, these results underscore the prognostic relevance of spatially resolved immune cell composition in patients with LAGC. The enrichment of cytotoxic and memory T cells, along with the distinct spatial pattern of Tregs, highlights the potential of immune contexture as a predictive biomarker for nICT response.

### 3.5 Prognostic value of candidate biomarkers and correlation assessment of immune infiltration

**Figure 5:**
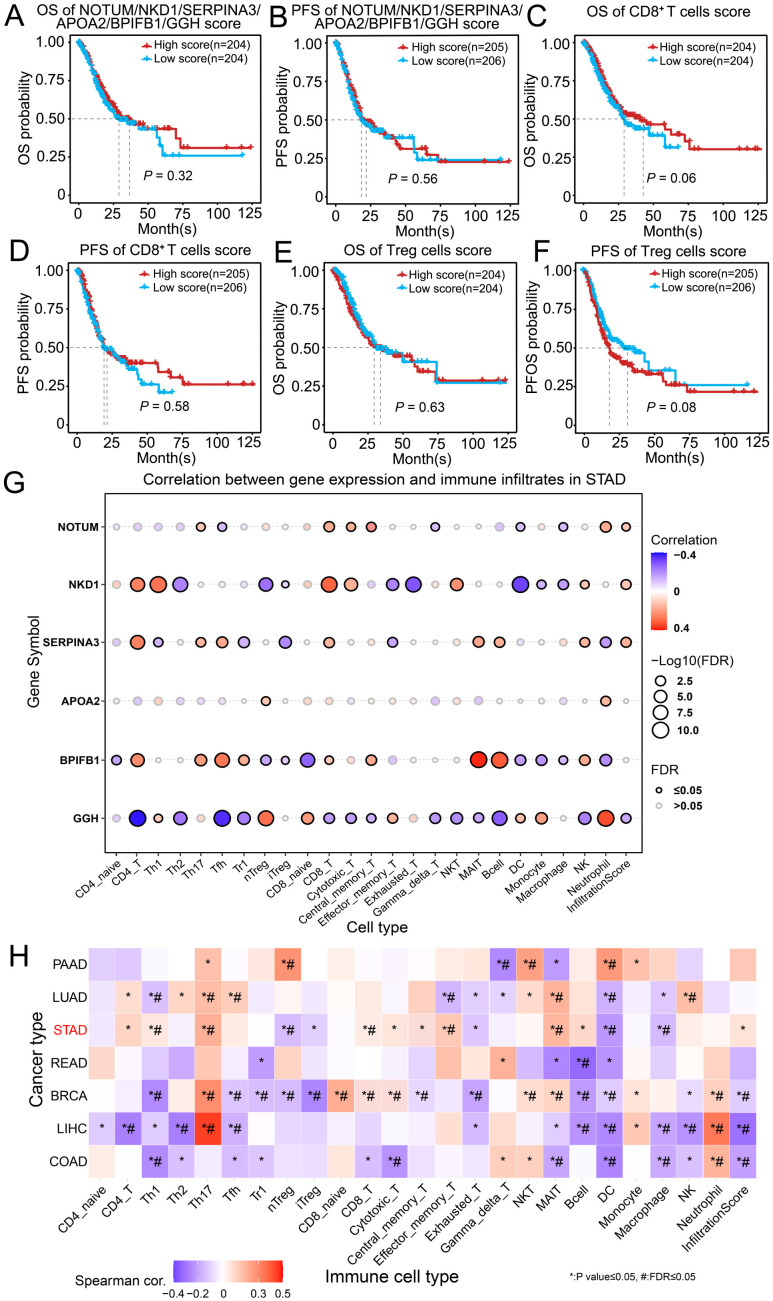
Prognostic and immunomodulatory associations of spatial signatures in public cohorts. **(A–F)** Kaplan–Meier curves for overall survival [OS; **(A, C, E)**] and progression-free survival [PFS; **(B, D, F)**] stratified by high/low expression of the six-gene signature (NOTUM/APOA2/SERPINA3/NKD1/GGH/BPIFB1) **(A, B)**, the CD8^+^ T cell signature **(C, D)**, and the regulatory cell (Treg) signature **(E, F, G)** Correlation matrix between individual gene expression and immune infiltration scores in patients with STAD. **(H)** Heatmap of correlations between the six-gene signature and immune cell infiltration in six cancer types. The color scale indicates the correlation coefficient, and asterisks denote statistical significance (*P < 0.05). PAAD, pancreatic adenocarcinoma; LUAD, lung adenocarcinoma; READ, rectal adenocarcinoma; BRCA, breast cancer; LIHC, liver hepatocellular carcinoma; COAD, colon adenocarcinoma. (P value ≤ 0.05, *; FDR ≤0.05, #).

To evaluate the clinical relevance of our spatial findings, we generated a six-gene signature (NOTUM, APOA2, SERPINA3, NKD1, GGH, and BPIFB1) from the most significant response-associated genes (FDR < 0.05, |log2FC| > 1) identified across the spatial transcriptomic analyses. These genes were enriched in responders in the pseudobulk analysis and in region-specific analyses of the TC and OTHER compartments, suggesting a coordinated response-associated program across spatial niches. We next assessed the prognostic and immunological relevance of this integrated signature in the TCGA-STAD cohort. Kaplan-Meier analysis revealed that compared with patients with low expression, patients with high expression of this six-gene signature tended to have longer OS and PFS (**Figures 5A, B**). These findings suggest a potentially favorable prognostic role for this gene set inpatients with GC. To further assess the spatial immunological relevance of this signature, we examined the correlation between the TC six-gene signature and immune-related signatures in the MA compartment. The TC six-gene signature showed a positive association with the MA CD8^+^ T-cell signature and a negative association with the MA Treg signature (**Supplementary Figure 6A**), suggesting that tumor-intrinsic transcriptional states are linked to the immune contexture of adjacent microenvironmental regions. We next examined the prognostic impact of immune context using gene signatures representative of CD8^+^ T cells and Tregs. A high CD8^+^ T cell signature score was associated with significantly improved OS (P = 0.06, borderline significance) and a strong trend toward better PFS (**Figures 5C, D**). Conversely, a high Treg signature score correlated with a notable trend toward shorter PFS, although no significant difference was observed for OS (**Figures 5E, F**). These findings reinforce the critical, opposing roles of cytotoxic and regulatory T cell populations in shaping clinical outcomes in patients with GC. To elucidate the potential mechanism linking our candidate genes to the immune microenvironment, we performed correlation analysis between individual gene expression and immune cell infiltration estimates in the TCGA-STAD cohort (**Figure 5G**). Notably, the expression of NOTUM, NKD1, and SERPINA3—the core tumor-derivedbiomarkers from our spatial analysis—was significantly positively correlated with overall immune infiltration and CD8^+^ T cell abundance but was negatively correlated with immunosuppressive iTreg infiltration. This pattern perfectly aligns with the immune-favorable microenvironment observed in nICT responders. To further explore this association, we analyzed the correlations between NOTUM, NKD1, and SERPINA3 expression and selected chemokines/receptors related to CD8^+^ T-cell or Treg recruitment in the TCGA-STAD cohort (**Supplementary Figure 6B**). Higher expression of these genes was positively correlated with CXCR3 and CCR5, but negatively correlated with CCL22 and CCR4, supporting their association with an immune-favorable microenvironment characterized by enhanced cytotoxic T-cell trafficking and reduced Treg-related signals. Finally, to assess the generalizability of the immune correlation pattern associated with our six-gene signature, we analyzed its relationship with immune cell infiltration across six additional cancer types (PAAD, LUAD, READ, BRCA, LIHC, and COAD) (**Figure 5H**). Strikingly, a conserved correlation pattern was observed, most prominently in STAD and BRCA. High signature expression was consistently associated with increased infiltration of CD8^+^ T cells, cytotoxic T cells, and memory T cells and decreased infiltration of Tregs. This cross-cancer conservation suggests that the biological interplay between this gene signature (encompassing the WNT inhibitor NOTUM/NKD1 and the serine protease inhibitor SERPINA3) and an antitumor immune context may be a fundamental axis influencing tumor–immune interactions beyond those in GC.

### 3.6 Independent clinical validation of spatially resolved biomarkers predicting nICT response

**Figure 6:**
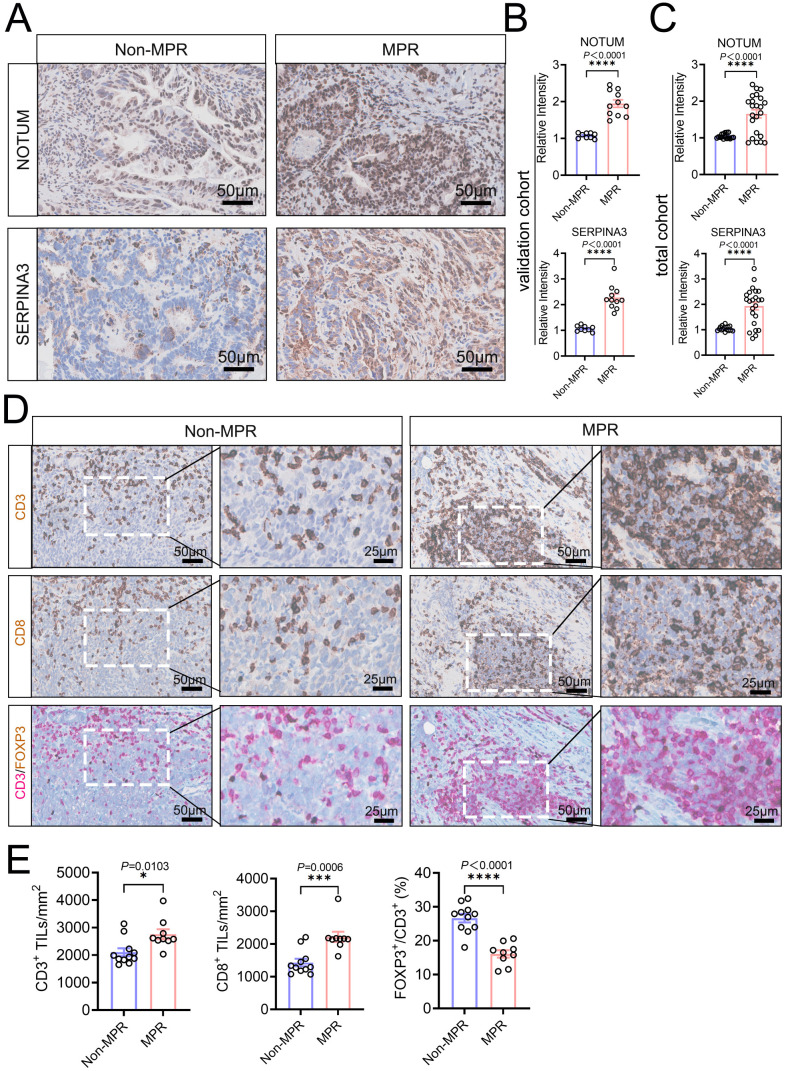
Independent clinical validation of protein biomarkers for predicting nICT response. **(A)** Representative IHC images of NOTUM and SERPINA3 staining in tumor regions from MPR and non-MPR patients in the validation cohort. Scale bar, 50 µm. **(B, C)** Quantification of NOTUM and SERPINA3 protein expression in tumor areas in the validation **(B)** and total **(C)** cohorts. **(D)** Representative IHC images of CD3, CD8, and FOXP3 staining in sequential sections from MPR and non-MPR patients. Scale bar, 100 µm. **(E)** Quantification of CD3^+^ and CD8^+^ T cell densities and the FOXP3^+^/CD3^+^ Treg ratio in the validation cohort (*P* < 0.05, *; *P* < 0.0001, ***).

To translate our spatially-derived transcriptional signatures into clinically applicable protein biomarkers, we performed immunohistochemical (IHC) validation in an independent cohort of 20 LAGC patients treated with nICT (validation cohort: MPR, n=9; non-MPR, n=11). The protein expression of NOTUM and SERPINA3 was significantly greater in the TCs of MPR patients than in those of non-MPR patients in the validation cohort, total cohort (**Figures 6A–C**) and discovery cohort (**Supplementary Figures 7A, B**), consistent with the DSP-derived mRNA findings, and confirming that the high pretreatment expression of these tumor-intrinsic proteins is a robust indicator of nICT sensitivity. We next quantified the preexisting immune context. MPR patients exhibited significantly greater densities of CD3^+^ and CD8^+^ T cells within the tumor microenvironment (**Figures 6D, E**; **Supplementary Figures 7C, D**). Moreover, the ratio of immunosuppressive regulatory T cells (Tregs, FOXP3^+^) to total T cells (CD3^+^) was markedly lower in MPR patients (**Figures 6D, E**; **Supplementary Figures 7C, D**). This inverse relationship between cytotoxic T cell abundance and Treg frequency alignswith the immune-favorable landscape associated with treatment response. ROC curve analysis showed that SERPINA3 had slightly better discriminative ability for predicting MPR than NOTUM, with AUCs of 0.803 and 0.765, respectively. The optimal cutoff values were 1.394 for SERPINA3 and 1.316 for NOTUM (**Supplementary Figure 7E**). In summary, these results demonstrate that high pretreatment protein expression of NOTUM and SERPINA3, increased CD8^+^ T cell infiltration, and a lower Treg cell ratio in biopsy tissues are favorable factors associated with an effective nICT response in patients with LAGC.

## 4 Discussion

NICT has emerged as a promising therapeutic strategy for patients with LAGC. However, a substantial proportion of patients derive limited clinical benefit, underscoring the urgent need to identify predictive biomarkers to guide patient stratification and personalized treatment. Conventional biomarker detection methods often rely on bulk tissue sequencing, which may overlook spatial heterogeneity within the TME, thereby compromising their predictive accuracy. DSP technology enables the resolution of molecular features within and around tumor regions, capturing intersample variations and enhancing the precision of spatial characterization of key molecular events (such as immune cell composition and tumor–stromal crosstalk). Leveraging automated segmentation, the GeoMx DSP platform facilitates compartment-specific expression profiling without the need for extensive tissue manipulation or manual tumor boundary annotation. This study demonstrates the potential of DSP in delineating the spatial architecture of the TME in LAGC patients and in identifying robust biomarkers that are predictive of nICT response.

Using DSP, we partitioned the TME into functionally distinct regions—tumor cell-enriched (TC), immune cell-enriched (MA), and stromal (OTHER) compartments, each of which exhibited unique transcriptional programs. In an independent LAGC cohort, when DSP was combined with IHC, we verified that the overexpression of NOTUM and SERPINA3 in TC regions was significantly associated with nICT sensitivity. Moreover, the MPR group showed a marked increase in CD8^+^ T cell infiltration and a decrease in Treg cell numbers in OTHER regions. Further analysis based on the STAD dataset in the TCGA public database revealed significant clinical associations between NOTUM, SERPINA3, and CD8^+^ T cells and Treg cells and the efficacy of nICT.

Our study revealed pronounced spatial heterogeneity in biomarker expression between the tumor core and peripheral regions, suggesting dynamic cross-compartment interactions and providing new insight into TME biomarker discovery. A key finding is that increased expression of NOTUM/NKD1 and SERPINA3 in tumor regions may serve as a predictive biomarker for nICT response and favorable clinical outcomes. NOTUM, a secreted member of the α/β hydrolase superfamily, functions as a palmitoleoyl-protein carboxylesterase that inactivates WNT ligands through depalmitoylation, thereby suppressing both canonical and noncanonical WNT signaling pathways (32, 36). In colorectal cancer, dysregulated NOTUM expression is closely correlated with cancer stem cell properties, cell proliferation activity and malignant biological behaviors, and it governs colorectal cancer progression by negatively controlling the hyperactivated Wnt/β-catenin signaling axis (37). Additionally, the expression level and enzymatic activity of NOTUM are fine-tuned by multiple regulatory layers, covering transcriptional regulation, epigenetic modification and post-translational modulation, further confirming its critical involvement in tumor initiation and progression (38). Collectively, the well-elucidated tumor-regulatory roles of NOTUM in various human cancers strongly support the rationality and clinical translational value of exploring its expression profile, functional phenotype and underlying molecular mechanism in gastric cancer, which is also the core innovative point of the present study. NKD1, a cytosolic protein, inhibits WNT signal transduction by binding and suppressing Disheveled (DVL). The WNT/β-catenin pathway is known to suppress T cell infiltration and reduce sensitivity to immunotherapy, potentially by modulating immune cells such as Tregs within the TME. Recent studies have further strengthened the concept that aberrant WNT/β-catenin activation promotes immune exclusion in gastric cancer and may contribute to resistance to immunotherapy. In particular, Li et al. demonstrated that β-catenin activation in gastric cancer tissues was negatively correlated with CD8^+^ T-cell infiltration, and that disruption of WNT/β-catenin signaling improved the sensitivity of gastric cancer cells to PD-1 blockade by enhancing T-cell activity (39). Mechanistically, this suggests that inhibition of WNT signaling may help remodel the tumor immune microenvironment and restore responsiveness to immune checkpoint therapy. Consistently, Ji et al. identified CCL28 as a direct transcriptional target of β-catenin/TCF and showed that β-catenin–induced CCL28 promoted regulatory T cell (Treg) recruitment, thereby facilitating gastric cancer progression through an immunosuppressive microenvironment (40). These findings indicate that WNT pathway antagonism may enhance antitumor immunity not only by increasing effector T-cell infiltration, but also by reducing Treg-cell–mediated immune suppression. More recently, Zhu et al. reported that circATM suppresses Wnt/β-catenin signaling in gastric cancer cells by binding PARP1, disrupting the PARP1/β-catenin/TCF4 complex, and inducing cell cycle arrest (41). Although this study primarily focused on tumor cell proliferation, it provides additional evidence that upstream suppression of WNT signaling can exert multifaceted antitumor effects and may represent a promising strategy to improve the efficacy of emerging gastric cancer therapies. As key negative regulators of WNT signaling, NOTUM and NKD1 may contribute to an immune-favorable contexture in LAGC. Our results suggest that NOTUM may sensitize LAGC to PD-1 blockade. High NOTUM expression in tumor regions was associated with suppressed WNT/β-catenin signaling (KEGG enrichment, **Figure 3B**) and enhanced antigen presentation mechanisms, which is consistent with preclinical evidencethat WNT inhibition promotes T cell infiltration and reduces Treg accumulation. Supporting this interpretation, additional analyses in the TCGA-STAD cohort showed that higher expression of NOTUM, NKD1, and SERPINA3 was associated with increased expression of CXCR3 and CCR5 and decreased expression of CCL22 and CCR4 (**Supplementary Figure 6**), a pattern consistent with enhanced CD8^+^ T-cell-related trafficking and reduced Treg-related signals. However, these observations remain correlative and should not be interpreted as direct evidence that NOTUM or related pathways causally regulate chemokine expression or immune-cell recruitment in gastric cancer. Further mechanistic studies are needed to determine whether NOTUM-mediated WNT inhibition directly contributes to immune remodeling in this setting. SERPINA3, a serine protease inhibitor, likely contributes to TME regulation and extracellular matrix remodeling through the inhibition of target proteases such as cathepsin G. Its elevated expression in several cancers (e.g., prostate and colorectal cancer) suggests a role in modulating immune-related proteolytic activity in the TME. In conclusion, NOTUM/SERPINA3 was associated with good prognosis in multiple cohorts, including the TCGA cohort and the validation cohort, supporting its value as a potential pancancer biomarker.

Previous studies have explored predictive biomarkers for nICT in gastric cancer from different perspectives, including systemic inflammatory indices, single protein markers, and multi-omic immune remodeling signatures. For example, IBI(Inflammatory burden index) was proposed as a convenient and cost-effective prognostic marker (42), while CD8A and PGF were suggested as candidate protein biomarkers linked to treatment response (43). In addition, multi-omic studies have shown that nICT induces profound immune remodeling, particularly with increased cytotoxic lymphocyte infiltration and reduced immunosuppressive features in MPR cases (44, 45). However, most of these studies either rely on peripheral indicators, focus on single markers, or emphasize post-treatment changes, and do not fully capture the spatial heterogeneity of the pretreatment tumor microenvironment. In contrast, our study integrates spatial transcriptomics with independent IHC validation to identify NOTUM, NKD1, SERPINA3, CD8^+^ T cell density, and Treg/CD3^+^ ratio as spatially resolved and clinically actionable predictors of nICT response.

Several limitations of this study should be acknowledged. First, the sample size was relatively small, which may limit the statistical power and generalizability of the findings. Second, the present study was primarily based on bioinformatic analyses and clinical correlations, and no functional experiments were performed to validate the biological role of NOTUM and SERPINA3. Therefore, their involvement in the regulation of CD8^+^ T-cell infiltration remains inferential and requires further confirmation through *in vitro* and *in vivo* studies. Third, the absence of external multicenter validation is another limitation, and the reproducibility and applicability of the proposed model in independent cohorts remain to be established. Future studies with larger, multicenter datasets and mechanistic validation are warranted to strengthen these findings.

Beyond tumor-derived signals, immune cell composition, especially the abundance of CD8^+^ T cells and the ratio of Tregs in OTHER regions, was strongly correlated with nICT response (46, 47). This finding aligns with established paradigms in cancer immunotherapy, wherein baseline CD8^+^ T cell infiltration is generally associated with an improved response to immune checkpoint inhibitors (48, 49). Importantly, our spatial analysis revealed that these immune cells are not uniformly distributed; instead, their localization within specific TME microdomains significantly influences treatment outcomes. The inverse correlation between Treg abundance and response further underscores the importance of overcoming immunosuppressive mechanisms to achieve therapeutic benefit. The spatial relationship between CD8^+^ T cells and Tregs highlights the potential utility of immune context-based stratification for LAGC patients receiving nICT (**Figure 6**). Moreover, the cross-compartment correlation between the TC six-gene signature and MA immune signatures suggests that this tumor-associated transcriptional program is linked to a favorable immune state at the tumor–immune interface. Another notable finding was the conserved association between a six-gene signature (NOTUM, APOA2, SERPINA3, NKD1, GGH, and BPIFB1) and immune infiltration across multiple cancer types, such as breast (BRCA), lung (LUAD), and colorectal (COAD) adenocarcinomas. These findings suggest that WNT pathway antagonism (via NOTUM/NKD1) and SERPINA3 expression may collectively sensitize diverse tumors to checkpoint inhibition.

## 5 Conclusion

In summary, our study identified NOTUM, SERPINA3, and CD8^+^ T cell density as spatially resolved predictors of response to neoadjuvant immunochemotherapy in locally advanced gastric cancer, linking tumor-intrinsic WNT pathway antagonism and SERPINA3 expression to a favorable immune contexture characterized by elevated CD8^+^ T cell infiltration and reduced Treg abundance. The translation of these spatial biomarkers into a clinically applicable immunohistochemistry-based assay using routine biopsy specimens offers a practical strategy for optimizing patient selection for nICT. These findings underscore the critical role of spatial architecture in biomarker discovery and open new avenues for precision immunotherapy in gastric cancer and potentially other malignancies.

## Data Availability

The datasets presented in this study can be found in online repositories. The names of the repository/repositories and accession number(s) can be found below: https://www.ncbi.nlm.nih.gov/, PRJNA1432121.
