# Supplementary figures and images for: Characterization of the tumor microenvironment in locally advanced gastric cancer and identification of spatially predictive biomarkers associated with beneficial neoadjuvant immunochemotherapy
Source: Front Immunol. 2026 May 14;17:1823308. doi: 10.3389/fimmu.2026.1823308 (PMC13215860; doi:10.3389/fimmu.2026.1823308)

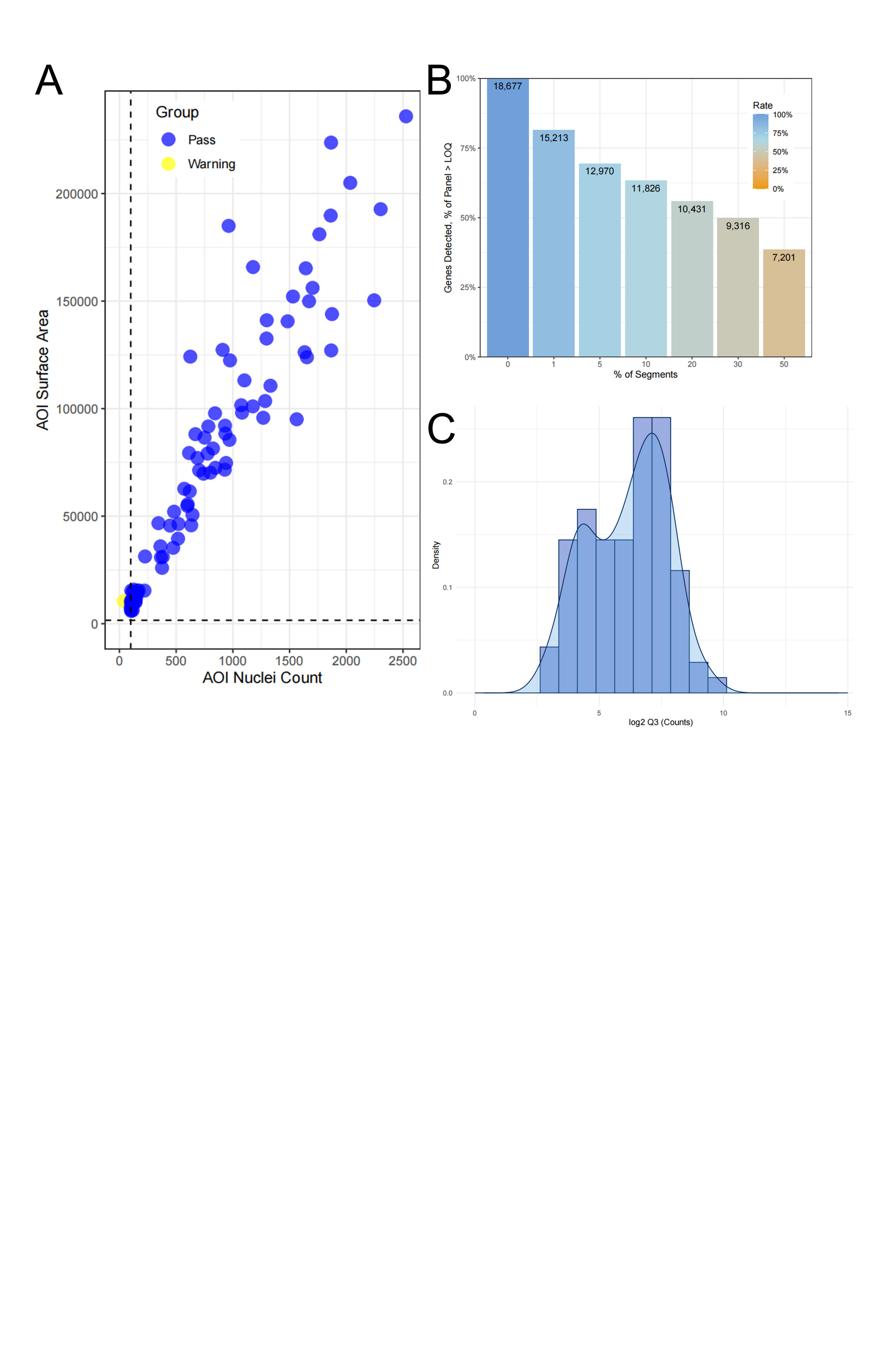

Supplement: Supplementary file 1 [file Image1.jpeg]

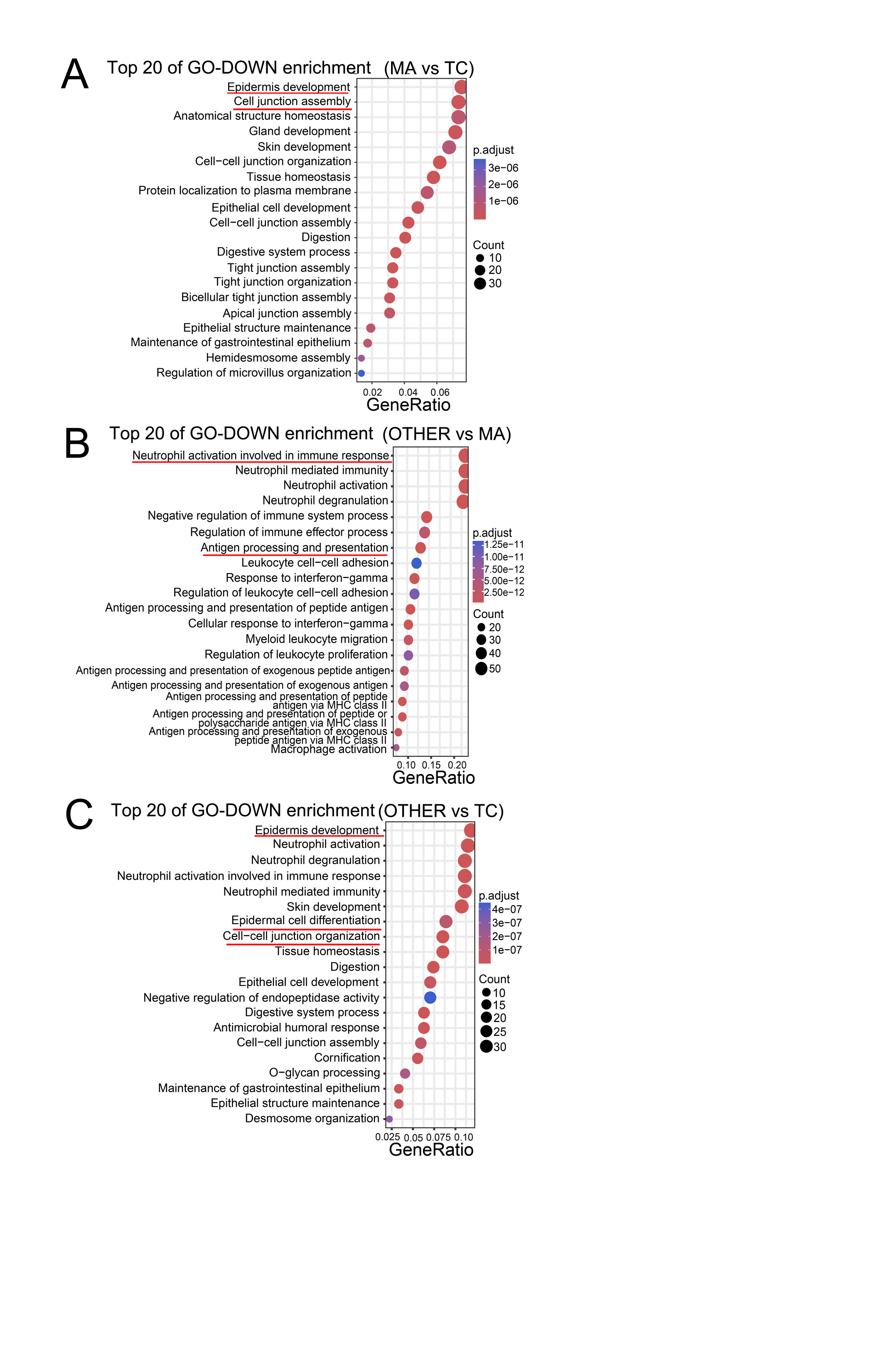

Supplement: Supplementary file 2 [file Image2.jpeg]

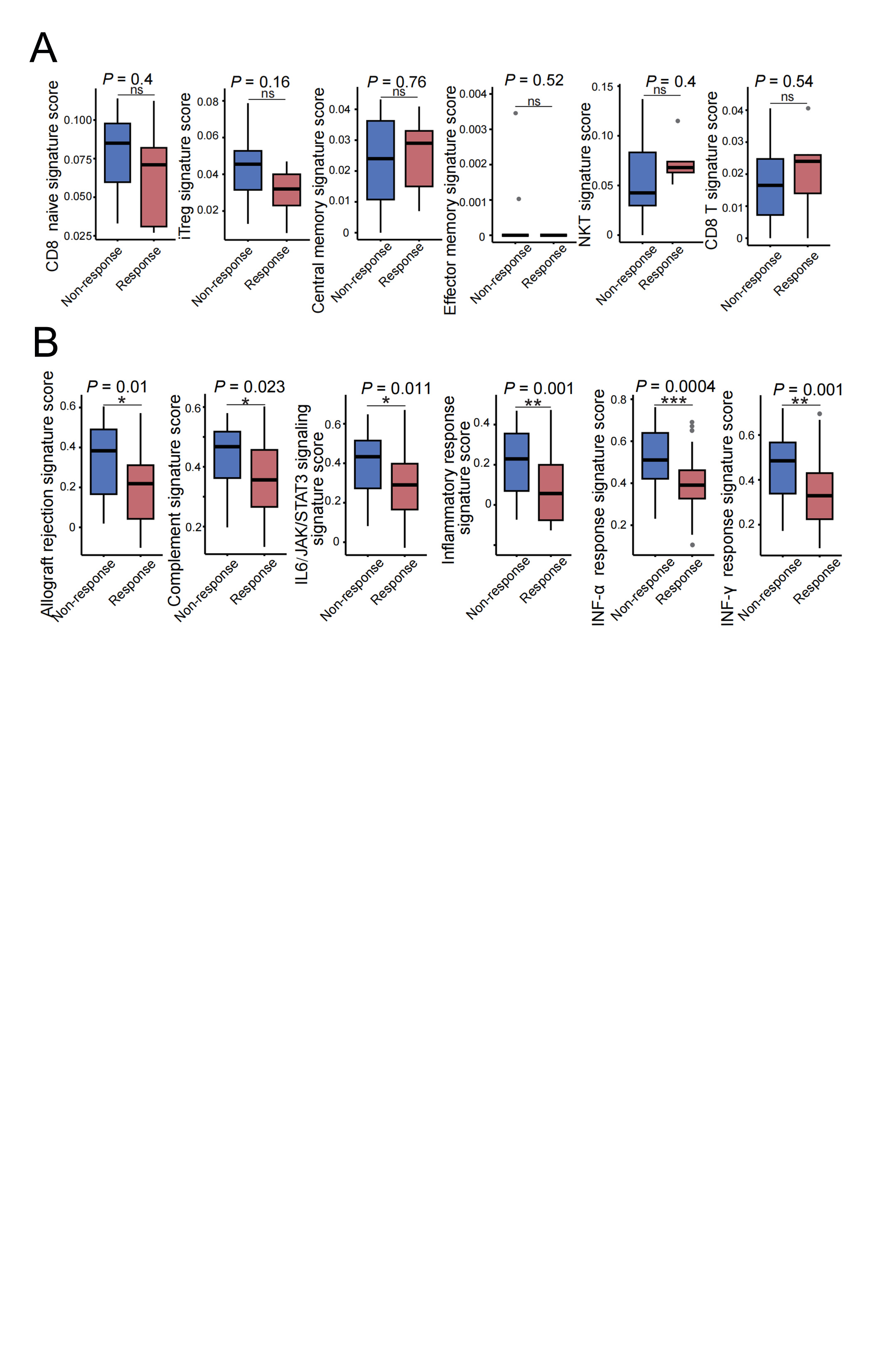

Supplement: Supplementary file 3 [file Image3.jpeg]

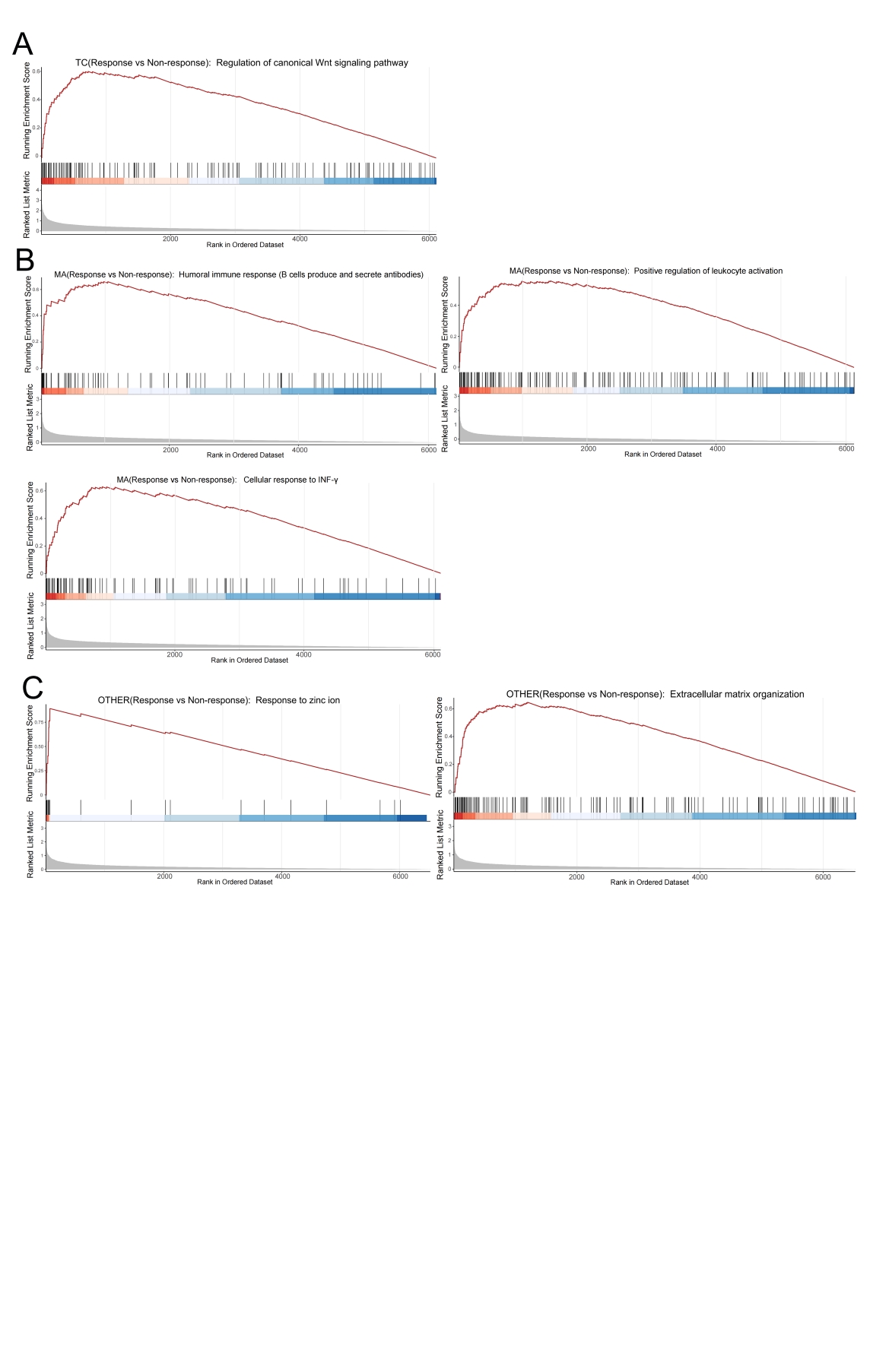

Supplement: Supplementary file 4 [file Image4.jpeg]

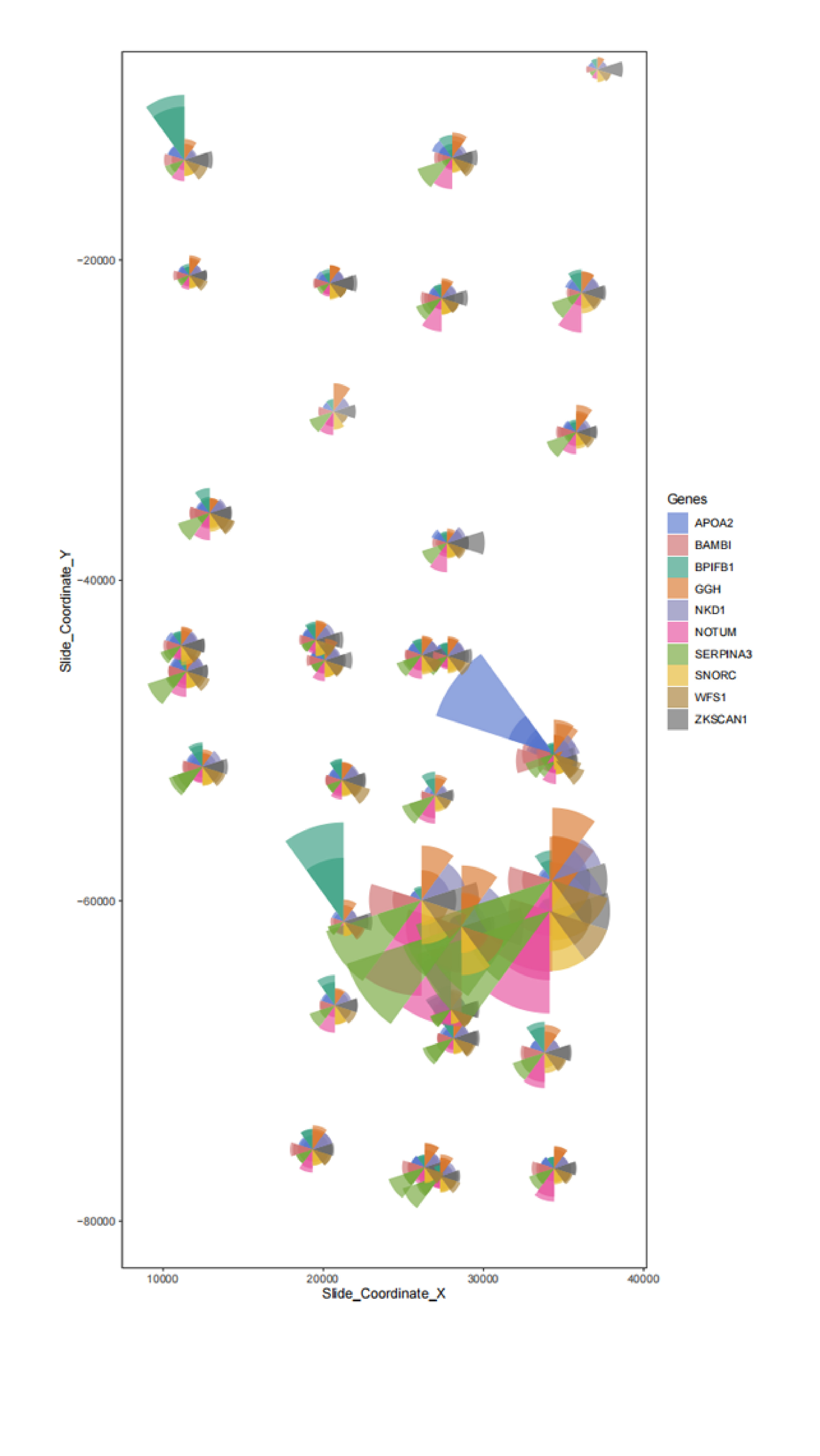

Supplement: Supplementary file 5 [file Image5.jpeg]

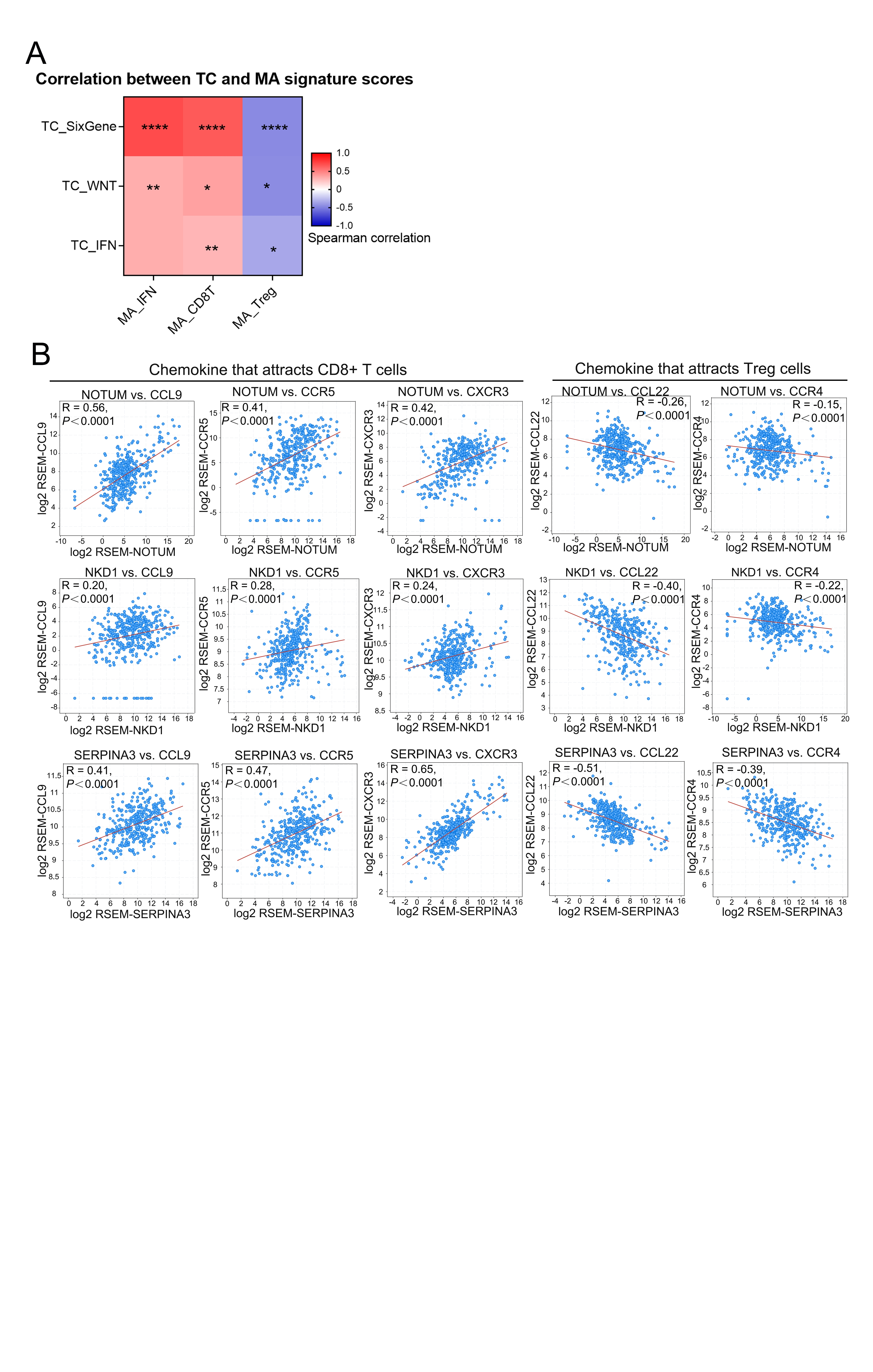

Supplement: Supplementary file 6 [file Image6.jpeg]

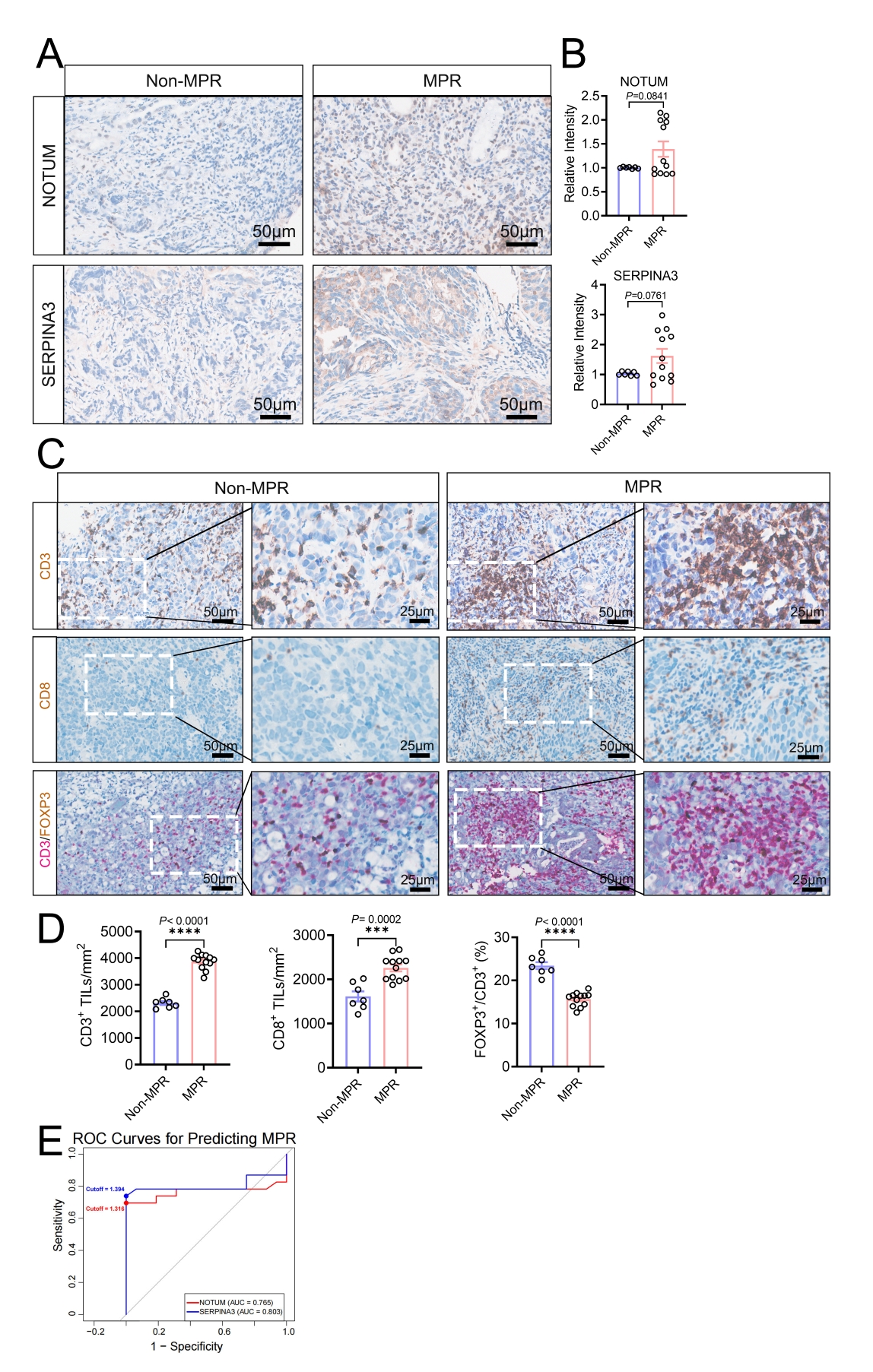

Supplement: Supplementary file 7 [file Image7.jpeg]
